# Validation and prognostic value of EZ-ALBI score in patients with intermediate-stage hepatocellular carcinoma treated with trans-arterial chemoembolization

**DOI:** 10.1186/s12876-022-02366-y

**Published:** 2022-06-14

**Authors:** Prooksa Ananchuensook, Supachaya Sriphoosanaphan, Sirinporn Suksawatamnauy, Nipaporn Siripon, Nutcha Pinjaroen, Nopavut Geratikornsupuk, Stephen J. Kerr, Kessarin Thanapirom, Piyawat Komolmit

**Affiliations:** 1 Division of Gastroenterology, Department of Medicine, Faculty of Medicine, Chulalongkorn University, Rama IV road, Pathumwan, Bangkok, 10330 Thailand; 2 Center of Excellence in Liver Diseases, King Chulalongkorn Memorial Hospital, Thai Red Cross Society, Bangkok, Thailand; 3 Liver Fibrosis and Cirrhosis Research Unit, Chulalongkorn University, Bangkok, Thailand; 4 Department of Radiology, Faculty of Medicine, Chulalongkorn University, Bangkok, Thailand; 5 Department of Medicine, Queen Savang Vadhana Memorial Hospital, Chonburi, Thailand; 6 Center of Excellence in Biostatistics, Faculty of Medicine, Chulalongkorn University, Bangkok, Thailand

**Keywords:** Easy albumin-bilirubin score, Intermediate-stage hepatocellular carcinoma, Trans-arterial chemoembolization, Overall survival

## Abstract

**Background:**

Heterogeneity of liver function and tumor burden in intermediate-stage hepatocellular carcinoma (HCC) results in different outcomes after transarterial chemoembolization (TACE). Easy albumin-bilirubin (EZ-ALBI), a simplified albumin-bilirubin (ALBI) score, has recently been proposed as a new prognostic score for HCC. This study aimed to validate the EZ-ALBI score and evaluate the impact of dynamic changes in patients with intermediate-stage HCC undergoing TACE.

**Methods:**

All patients with HCC treated with TACE at King Chulalongkorn Memorial Hospital, Bangkok, Thailand, between January 2015 and December 2019 were prospectively enrolled. Intermediate-stage HCC was defined as Barcelona Clinic Liver Cancer (BCLC) stage B or unresectable single HCC with size > 5 cm in BCLC stage A. EZ-ALBI and ALBI scores were calculated and stratified into three different grades. Overall survival (OS) and prognostic factors were assessed using the Kaplan–Meier curve and Cox proportional hazard model. Decision analysis curves were used to evaluate the clinical utility of the predictive scores.

**Results:**

Among 672 patients with HCC treated with TACE, 166 patients with intermediate-stage HCC who met the eligibility criteria were enrolled. The median OS of all patients in the cohort was 21 months. A good correlation between the EZ-ALBI and ALBI scores was observed (correlation coefficient 1.000, *p* < 0.001). The baseline EZ-ALBI grades 1, 2, and 3 were 24.5%, 70%, and 5.5%, respectively. EZ-ALBI grade can stratify patients with significantly different prognoses (*p* = 0.002). Baseline EZ-ALBI grade 2, 3, and serum alpha-fetoprotein > 20 ng/ml were significantly associated with OS [hazard ratio (HR) 2.20 (95% confidence interval [CI] 1.24–3.88, *p* = 0.007), 3.26 (95% CI 1.24–8.57, *p* = 0.016), and 1.77 (95% CI 1.10–2.84, *p* = 0.018), respectively]. Following TACE, 42 (29.6%) patients had a worsening EZ-ALBI grade. However, the EZ-ALBI grade migration was not significantly correlated with OS. EZ-ALBI and ALBI score provided improved discriminatory ability (Harrell’s concordance index 0.599 and 0.602, respectively) and better net benefit compared with Child-Turcotte-Pugh and Model for End-stage Liver Disease scores.

**Conclusions:**

The baseline EZ-ALBI score demonstrated good predictive performance for survival and a strong correlation with conventional ALBI scores. Both the EZ-ALBI and ALBI scores outperformed other prognostic models in patients with intermediate-stage HCC receiving TACE. However, the dynamic change in the EZ-ALBI grade after TACE was not associated with postprocedural survival.

## Background

Hepatocellular carcinoma (HCC) is a leading cause of death worldwide. It ranked as the sixth most common cancer with more than 80,000 deaths annually [1]. Chronic hepatitis B virus (HBV) infection is the major cause of HCC in Eastern countries, while chronic hepatitis C virus (HCV) infection and non-alcoholic fatty liver disease are the most common etiologies among HCC patients in the west [2–4]. Despite recent advanced treatments, HCC is still associated with high mortality rate [5].

Current guidelines recommend treatment algorithms according to the Barcelona Clinic Liver Cancer (BCLC) staging [2–4].Trans-arterial chemoembolization (TACE) is recognized as a standard therapeutic option for intermediate-stage HCC. However, not all patients benefit from this treatment modality [6]. A significant proportion of patients have liver decompensation following the procedure, leading to poor clinical outcomes [7]. This could be explained by a variety of liver statuses and tumor burdens. The high heterogeneity of patients with intermediate-stage HCC ultimately results in various overall survival (OS) and treatment responses after TACE [8].

Unlike other cancers, the prognosis of HCC does not depend solely on the tumor burden. Background of chronic liver disease is also a crucial factor for survival in patients with HCC [9]. Various classifications of liver reserve function have been proposed for clinical decision-making and prediction of patient survival. The Child-Turcotte-Pugh (CTP) score is widely accepted as a standard tool for assessing liver function. However, arbitrarily defined cutoffs and clinically challenging assessment of hepatic encephalopathy and ascites could affect accurate prognostication [10]. Later, albumin-bilirubin (ALBI) score has emerged as an alternative score to assess liver impairment among patients with HCC [11]. The validation of ALBI score in several cohorts of patients with HCC, especially in the intermediate stage, showed a significant association with OS [12, 13]. ALBI grade migration after TACE also demonstrated adverse effects on patient survival [14]. However, the complexity of score calculation was a major drawback and limited its application in real-life practice. Therefore, an easy albumin-bilirubin (EZ-ALBI) score, a simplified and user-friendly formula for the ALBI score, has been developed [15]. EZ-ALBI score was significantly correlated with the conventional ALBI score and demonstrated good prognostic prediction across all HCC stages [15, 16]. With its simplicity, the EZ-ALBI score may potentially replace the ALBI score in clinical practice.

To date, no study has explored the role of the EZ-ALBI score as a pretreatment prognostic model for patients with HCC undergoing TACE. Additionally, no data exist on whether the dynamic change in the EZ-ALBI score after TACE could predict patient survival following the procedure. Therefore, our study aimed to validate the EZ-ALBI score and evaluate the impact of EZ-ALBI grade alterations on survival in patients with intermediate-stage HCC after treatment with TACE.

## Methods

### Patients and study design

Data of patients with HCC who underwent the first session of TACE at King Chulalongkorn Memorial Hospital (KCMH), a tertiary referral center and academic teaching hospital in Bangkok, Thailand, from January 2015 to December 2019 were retrospectively collected. According to the American Association for the Study of Liver Disease guidelines, HCC is diagnosed as an arterial-enhancing liver mass on the background of cirrhosis or chronic liver disease, using abdominal imaging or histopathology. Intermediate-stage HCC was defined as BCLC B or unresectable tumor with size > 5 cm in BCLC A, which represents the optimal candidates for TACE [6]. Patients aged > 18 years with preserved liver function defined by CTP A-B were included. The exclusion criteria were spontaneous tumor rupture, subsequent surgical resection after TACE, and concomitant treatment with other locoregional procedures or systemic therapies.

Baseline characteristics, including age, sex, etiologies of liver diseases, CTP score, and Eastern Cooperative Oncology Group status, were collected. Tumor characteristics, including tumor number, location, and size of HCC, were documented. Liver function test results and serum alpha-fetoprotein (AFP) levels were retrospectively recorded at baseline before TACE and 1–3 months after the procedure.

The formula for the ALBI score was 0.66 × log_10_bilirubin (µmol/L) – 0.0085 × albumin (g/L). The ALBI score was stratified into grades 1, 2,and 3 with scores of ≤ − 2.6, > − 2.6 to  − 1.39, and > − 1.39, respectively [11].The EZ-ALBI score was calculated as total bilirubin (mg/dL) – [9 × albumin (g/dL)] and subsequently stratified into three grades. EZ-ALBI grades 1, 2, and 3 were ≤ − 34.4, -34.4 to − 22.2, and > − 22.2, respectively [15]. The OS was calculated from the date of TACE to the death date recorded in the Thailand National Death Register. Patients who had not died were censored at their most recent follow-up.

### TACE procedure

Conventional TACE was performed by experienced interventional radiologists in the Division of Interventional Radiology, Department of Radiology, KCMH, Bangkok, Thailand. The right common femoral artery was accessed using the Seldinger technique, followed by installation of a 5F introducer sheath. Routinely, a 5F Yashiro catheter in conjunction with a 0.035″ Terumo guide wire was used to perform celiac and superior mesenteric artery (SMA) angiograms, CT arterial portography via the SMA, and CT hepatic angiography via the hepatic artery (mostly at the common hepatic artery, but sometimes at the hepatic artery proper or right or left hepatic artery). After selective catheterization of the tumor feeders was achieved, chemoembolization was performed until the feeders were occluded, using a mixture of 10 ml of Lipiodol, 20 mg of Mitomycin in 2 ml of sterile water, and 1–3 ml of contrast media with an intervening infusion of 500 mg of 5-Fluorouracil solution in the same proportion. Finally, gel foam embolization of the proximal feeder was performed. Repeated TACE may be scheduled for no less than 4 weeks, depending on the presence of residual or recurrent viable tumors.

## Statistical analysis

All statistical analyses were performed using the SPSS software (version 22.0; IBM Corp., NY, USA) and Stata 17 (Stata Corp, TX, USA). For baseline characteristics, categorical variables are presented as frequency (%), and continuous variables are shown as median and interquartile range. Categorical variables were compared using the chi-square test or Fisher’s exact test, whereas continuous variables were assessed using the Mann–Whitney *U*-test. Within patient pre- and post-TACE laboratory assessments were compared using a paired *t*-test. OS was assessed using Kaplan–Meier curves and Cox proportional hazard regression. Terms with univariable *P*-values < 0.05 were adjusted for in a multivariable model.

The EZ-ALBI scores assessed before and after TACE were externally validated in our population, and the correlation between EZ-ALBI and conventional ALBI scores was performed using regression analysis. Calibration plots were constructed by plotting the observed mortality versus the predicted probabilities in deciles of risk, and calibration was assessed using a Hosmer–Lemeshow goodness of fit test. Comparison with other prognostic scores, including the CTP and Model for End-stage Liver Disease (MELD) scores, was performed using the area under the receiver operating characteristic (AUROC) curve. The discrimination ability of the different prognostic scores was assessed using Harrell’s concordance index (C-index). The clinical net benefit of all prognostic scores was evaluated using decision curve analysis (DCA).

The study was reviewed and approved by the Ethics Committee and Institutional Review Board of the Faculty of Medicine, Chulalongkorn University, Bangkok, Thailand (IRB Number: 467/64). The study protocol was in accordance with the Declaration of Helsinki (1989) of the World Medical Association.

## Results

### Baseline characteristics

**Fig. 1:**
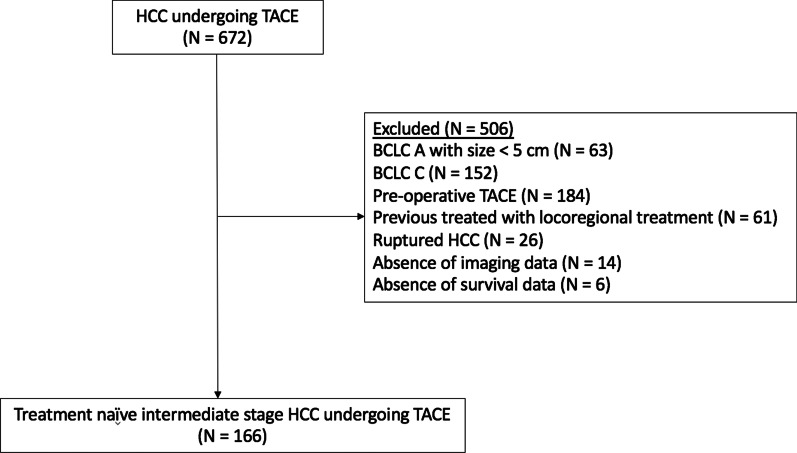
Flowchart of patient eligibility. BCLC, Barcelona clinic liver cancer; cm, Centimeter; HCC, Hepatocellular carcinoma; *N*, Number; TACE, Trans-arterial chemoembolization

Among 672 patients who underwent TACE at KCMH during the study period, 166 patients who met the inclusion criteria were enrolled (Fig. 1). The median follow-up time was 53 months (interquartile range [IQR] 37–62 months). Baseline clinical and tumor characteristics are summarized in Table 1. The majority of patients were male (76.5%), with a mean age of 65 ± 10.5 years. The common underlying etiologies of liver disease were HBV virus infection (39.8%) and HCV virus infection (25.9%). Overall, 156 (94%) patients had cirrhosis; 80.7% were classified as CTP A. Approximately 75% of patients had multiple HCC, and almost half (46.4%) had a maximal diameter of 3–7 cm. Ninety percent of the patients had unilobar disease, 45.8% in the right lobe, and 45.2% in the left lobe. Laboratory findings at baseline and 1–3 months after TACE are shown in Table 2. Following TACE, there was a statistically significant increase in total bilirubin (mean change from 1.03 ± 0.61 to 1.17 ± 0.86 mg/dL, *p* = 0.027), a reduction in albumin (mean change 3.50 ± 0.57 to 3.20 ± 0.56, *p* < 0.001), and an increase in INR (mean change from 1.16 ± 0.13 to 1.20 ± 0.13, *p* < 0.001).

**Table 1:** Baseline clinical and tumor characteristics

Baseline characteristics	*N* (%)/ median (IQR) (*N* = 166)
Sex (male)	127 (76.5%)
Age (years)	65 (57.75–72)
*Etiologies of liver disease *	
HBV infection HCV infection NASH Cryptogenic Alcohol AIH	73 (44.0%) 50 (30.1%) 16 (9.6%) 14 (8.4%) 12 (7.3%) 1 (0.6%)
*Cirrhosis 156(94.0%)*	
CTP A - Score 5 - Score 6 CTP B - Score 7 - Score 8	134 (80.7%) 87 (52.4%) 47 (28.3%) 22 (13.3%) 17 (10.2%) 5 (3.0%)
MELD score	8.88 (7.69 – 10.43)
*Number of HCC*	
1 2–3 > 3	49 (25.3%) 57 (34.4%) 67 (40.4%)
*Location of HCC *	
Both lobes Right lobe Left lobe	76 (45.8%) 75 (45.2%) 15 (9.0%)
*Largest tumor diameter (cm)*	
≤ 3 > 3—≤ 7 > 7—≤ 10 > 10	16 (9.6%) 77 (46.4%) 42 (25.3%) 31 (18.7%)

*AIH* Autoimmune hepatitis, *cm* Centimeter, *CTP* Child-Turcotte-Pugh, *HBV* Hepatitis B virus, *HCC* Hepatocellular carcinoma, *HCV* Hepatitis C virus, *IQR* Interquartile range, *MELD* Model for end-stage liver disease, *N* Number, *NASH* Non-alcoholic Steatohepatitis, *SD* Standard deviation

**Table 2:** Laboratory findings at baseline and after TACE

Laboratory findings	Baseline, Mean ± SD	Post-TACE, Mean ± SD	*p*-value
Total bilirubin (mg/dL)	1.03 ± 0.61	1.17 ± 0.86	0.027*
AST (U/L)	77.20 ± 51.65	73.69 ± 57.05	0.59
ALT (U/L)	56.21 ± 38.35	52.48 ± 43.95	0.300
Albumin (g/dL)	3.50 ± 0.57	3.20 ± 0.56	<0.001*
INR	1.16 ± 0.13	1.20 ± 0.13	<0.001*
Platelet (× 10^3^/µL)	167.46 ± 107.53	169.58 ± 116.82	0.62
Creatinine (mg/dL)	0.89 ± 0.36	0.88 ± 0.68	0.93
AFP (ng/mL)	3460.38 ± 14382.67	2449.96 ± 8.177.59	0.85

*AFP* Alpha fetoprotein, *ALT* Alanine aminotransferase, *AST* Aspartate aminotransferase, *INR* International normalized ratio, *mg/dL* Milligram per deciliter; ng/mL, Nanogram per milliliter; *SD* Standard deviation, *TACE* Trans-arterial chemoembolization, *U/L* Units per liter, *µL* Microliter

^*^p-value ≤ 0.05

### EZ-ALBI and ALBI score/grade at baseline and after TACE

The EZ-ALBI and ALBI scores at baseline and after TACE are shown in Table 3. Baseline total bilirubin and albumin evaluations were conducted a median of 1 (IQR 1–13.25) days before TACE, and median time for the second assessment was 35 (IQR 28.3–43.7) days after the procedure. A good correlation between the EZ-ALBI and ALBI scores at baseline and after TACE was observed, with correlation coefficients of 1.000 [95% confidence interval (CI) 0.983–1.000, *p* < 0.001] and 0.986 (95% CI 0.980–0.991, *p* < 0.001), respectively. At baseline, most patients had an EZ-ALBI grade of 2 (69.9%). Notably, there was no significant change in the EZ-ALBI and ALBI scores after TACE. Regarding EZ-ALBI grade migration, most patients (66.2%) remained at the same grade following TACE. However, approximately one-third of patients had worsening EZ-ALBI grades (Table 4). The proportions of patients with EZ-ALBI grades 1, 2, and 3 after TACE were 16 (11.0%), 105 (72.4%), and 9 (16.6%), respectively.

**Table 3:** EZ-ALBI and ALBI score/grade at baseline and after TACE

Scores and grade	Baseline (*N* = 163) *N* (%)/ mean ± SD	Post-TACE (*N* = 145) *N* (%)/ mean ± SD	*p*-value
EZ-ALBI score	− 37.70 ± 92.63	− 27.61 ± 5.51	0.185
*EZ-ALBI grade *			
-Grade 1 -Grade 2 -Grade 3	40 (24.5%) 114 (69.9%) 9 (5.5%)	16 (11.0%) 105 (72.4%) 24 (16.6%)	
ALBI score	− 2.89 ± 8.76	− 1.93 ± 0.59	0.181
*ALBI-grade*			
-Grade 1 -Grade 2 -Grade 3	40 (24.6%) 111 (68.0%) 12 (7.4%)	18 (12.4%) 95 (65.5%) 32 (22.1%)	

*ALBI* Albumin-bilirubin, *EZ-ALBI* Easy albumin-bilirubin, *N* Number, *SD* Standard deviation, *TACE* Trans-arterial chemoembolization

**Table 4:** EZ-ALBI and ALBI grade change after TACE

Grade change (*N* = 142)	EZ-ALBI, *N* (%)	ALBI, *N* (%)
Improving grade	6 (4.2%)	6 (4.2%)
Same grade	94 (66.2%)	92 (64.8%)
Worsening grade	42 (29.6%)	44 (31.0%)

*ALBI* Albumin-bilirubin, *EZ-ALBI* Easy albumin-bilirubin, *N* Number

### OS and factors associated with OS according to EZ-ALBI grade

**Fig. 2:**
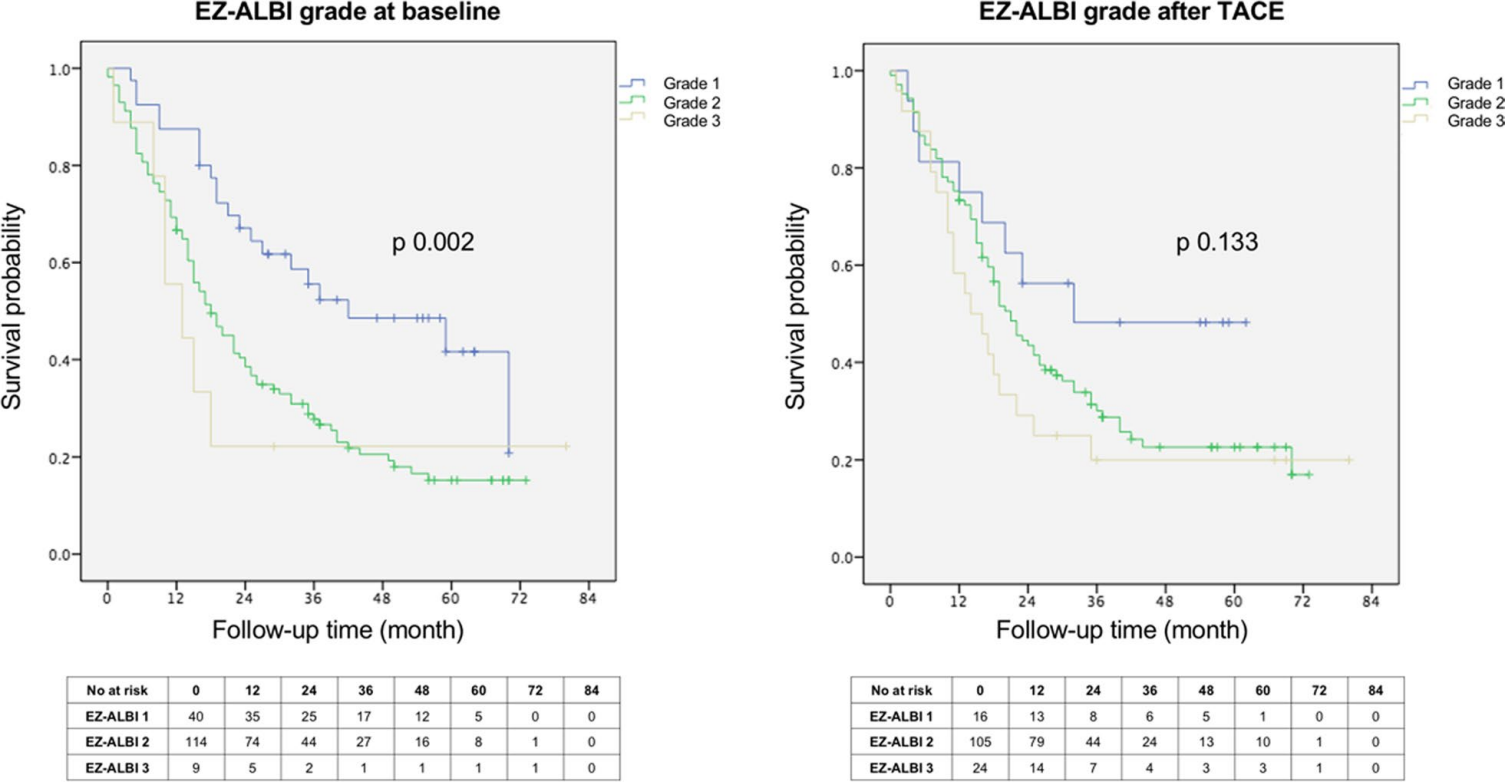
Kaplan–Meier curves showing overall survival probability according to EZ-ALBI grade at baseline (left panel) and after TACE (right panel) ALBI, albumin-bilirubin; EZ-ALBI, easy albumin-bilirubin; TACE, trans-arterial chemoembolization

The median OS of our cohort was 21 (95% CI 16.9–25.1) months. The survival distributions of patients stratified by EZ-ALBI grade at baseline and after TACE are shown in Fig. 2. The baseline EZ-ALBI grade stratified patients into three groups with significantly different survival rates. The median OS was 42 (95% CI 13.3–70.6) months, 18 (95%CI 13.7–22.2) months, and 13 months (95% CI 6.0–19.9) for EZ-ALBI grade 1, 2, and 3, respectively (logrank *p* = 0.002). The 1-year survival rates of patients with baseline EZ-ALBI grades 1, 2, and 3 were 87.5%, 64.9%, and 55.6%, respectively. In multivariate analysis, AFP > 20 ng/ml [hazard ratio (HR) 1.77 [95% CI 1.101–2.841, *p* = 0.018] and increasing baseline EZ-ALBI grade were significantly associated with OS. Compared to patients with EZ-ALBI grade 1, the HR for grade 2 was 2.20, [95% CI 1.24–3.88, *p* = 0.007], and 3.26 [95% CI 1.24–8.57, *p* = 0.016] for those with grade 3. (Table 5). Notably, EZ-ALBI grade migration after TACE did not seem to be related to posttreatment survival.

**Table 5:** Univariable and multivariable analysis of factors associated with survival in patients with HCC underwent TACE using Cox regression

Univariate analysis				Multivariate analysis			
Factors	HR	95%CI	*p*-value	Factors	HR	95%CI	*p*-value
Age	0.99	0.97–1.007	0.26				
Sex (male)	1.24	0.99–1.56	0.07				
MELD score	1.07	0.98–1.16	0.14				
Within up-to-seven criteria	0.92	0.68–1.26	0.62				
CTP B	1.17	0.69–1.99	0.57				
AFP > 20 ng/mL	1.82	1.16–2.85	0.009*	AFP > 20 ng/mL	1.77	1.10–2.84	0.018*
Baseline EZ-ALBI Grade 1 Grade 2 Grade 3		– 1.36–3.52 1.14–6.40	– 0.001* 0.023*	Baseline EZALBI Grade 1 Grade 2 Grade 3		- 1.24–3.88 1.24–8.57	- 0.007* 0.016*
	1 (ref) 2.19 2.71				1 (ref) 2.20 3.26		
Worsening EZ-ALBI grade	0.94	0.60–1.46	0.77				

*AFP* Alpha fetoprotein, *CI* Confident interval, *CTP* Child-Turcotte-Pugh, *EZ-ALBI* Easy albumin-bilirubin, *HR* Hazard ratio, *MELD* Model for end-stage liver disease, *ng/mL* Nanogram per milliliter

^*^Significant *p*-value is ≤ 0.05

### Comparison with other prognostic scores

**Fig. 3:**
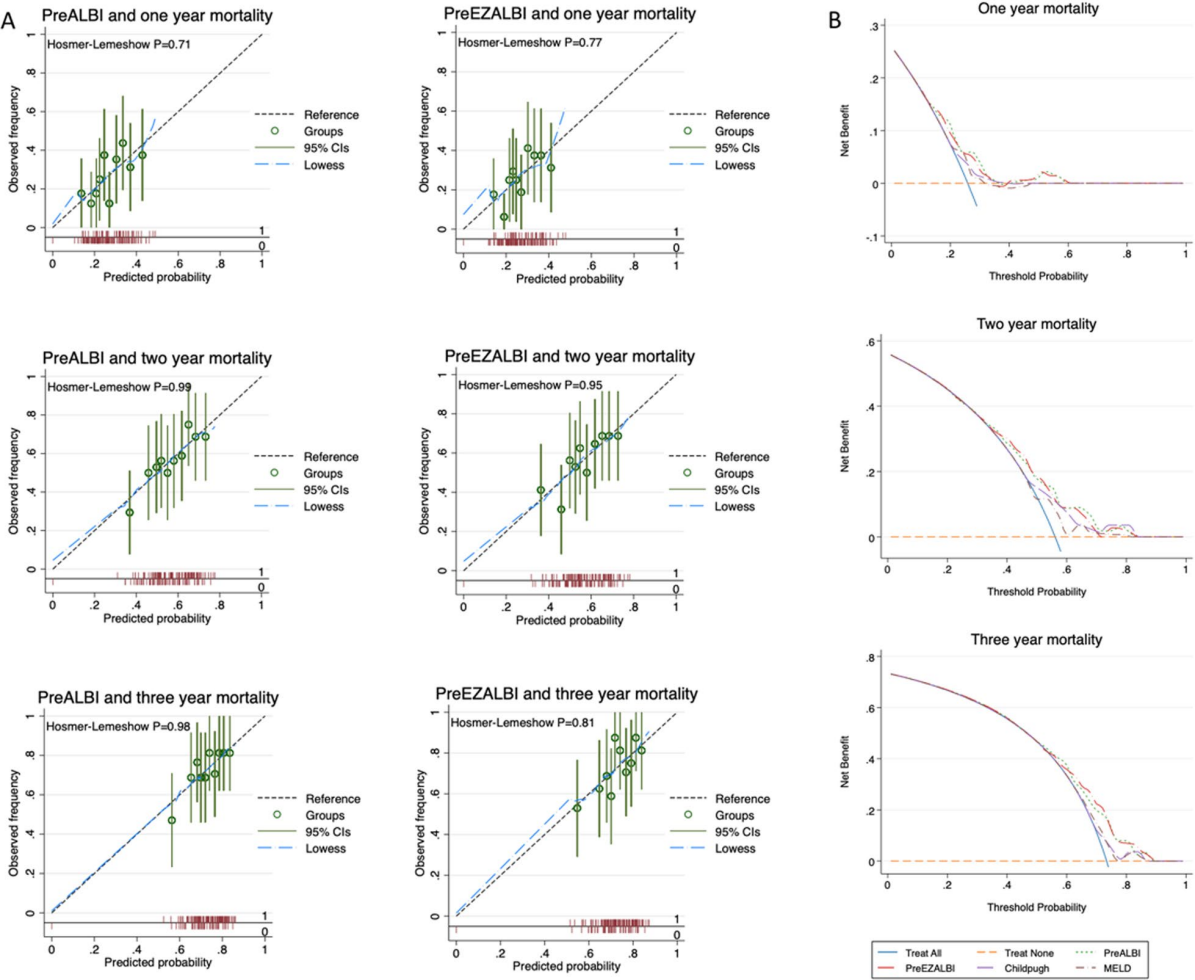
**A** Calibration plot of EZ-ALBI and ALBI scores for one-, two- and three-year mortality **B** Decision curve analysis for EZ-ALBI, ALBI, CTP, and MELD scores in terms of one-, two- and three-year mortality ALBI, albumin-bilirubin; CI, confident interval, EZ-ALBI albumin-bilirubin, MELD, model for end-stage liver disease.

The EZ-ALBI and ALBI scores showed good predictive performance represented by the calibration curves for one-, two- and three-year mortality (Fig. 3A); Hosmer–Lemeshow P-values ranged from 0.71 to 0.99 confirming the fit adequacy. The performances of the EZ-ALBI score and other prognostic scores, including the CTP, MELD, and ALBI scores, are shown in Table 6. The AUROCs of the EZ-ALBI score at baseline for predicting death at 1, 2, and 3 years were 0.613, 0.624, and 0.623, respectively. Although all mentioned scores include both albumin and bilirubin as component parameters, EZ-ALBI and ALBI scores showed higher AUROCs for prediction compared to those of CTP and MELD scores. Regarding discriminative ability, the EZ-ALBI and ALBI scores had higher Harrell's C-index than the CTP and MELD scores. DCA curves were drawn to assess the clinical practicability of the scores. Of note, the EZ-ALBI and ALBI scores demonstrated a similar trend of net benefit for 1-, 2-, and 3-year mortality. Both EZ-ALBI and ALBI scores also had better net benefit compared to CTP and MELD scores. (Fig. 3B).

**Table 6:** The AUROCs for predicting death of EZ-ALBI and other prognostic scores

Prognostic scores	AUROC for survival at 1 year	*p*-value	AUROC for survival at 2 year	*p*-value	AUROC for survival at 3 year	*p*-value	*C*-index
EZ-ALBI score	0.613 (0.517–0.709)	0.027	0.624 (0.537–0.711)	0.007	0.623 (0.525–0.720)	0.015	0.599 (0.542–0.656)
ALBI score	0.624 (0.529–0.720)	0.015	0.624 (0.537–0.711)	0.007	0.610 (0.513–0.707)	0.030	0.602 (0.545–0.659)
CTP score	0.557 (0.453–0.662)	0.276	0.575 (0.485–0.665)	0.110	0.524 (0.424–0.625)	0.637	0.554 (0.497–0.610)
MELD score	0.588 (0.488–0.689)	0.095	0.577 (0.486–0.669)	0.104	0.575 (0.469–0.680)	0.169	0.545 (0.486–0.604)

*ALBI* Albumin-bilirubin, *AUROC* Area under the receiver operating characteristic, *C*-index, Harrell’s concordance index, *CTP* Child-Turcotte-Pugh, *EZ-ALBI* Easy albumin-bilirubin, *MELD* Model for end-stage liver disease

## Discussion

The heterogeneity of patients with intermediate-stage HCC has raised several issues in the clinical practice. Diversity in tumor burden and liver function reserve exerts a crucial impact on the survival and clinical course of HCC [17]. A thorough evaluation of preserved liver status plays an important role in optimizing the patient’s health benefits after TACE. The present study demonstrated that the EZ-ALBI score, a simple liver reserve assessment, can stratify patients treated with TACE into distinct survival categories. Patients with pretreatment EZ-ALBI grades 2 and 3 had significantly decreased OS compared to those with EZ-ALBI grade 1. Almost 30% of the patients had worsening EZ-ALBI grades following TACE. However, EZ-ALBI grade migration was not associated with posttreatment survival.

Liver functional reserve is a significant predictor of survival after TACE [18]. Following TACE, deterioration of liver function is usually observed and ultimately affects OS [7, 19–21]. At one month after TACE, 30% of patients had higher serum bilirubin levels and more than half (52%) had lower albumin levels, indicating deteriorating hepatic function [7]. These changes also affected ALBI and EZ-ALBI grades. A study by Chi et al. demonstrated that 24.3% of patients had ALBI grade alterations at 1 month after TACE. In addition, ALBI grade migration to grade 3 was independently correlated with OS [14]. In our study, we also observed a significant increase in serum bilirubin after TACE together with a reduction in serum albumin. More than one-third of the patients in our cohort had EZ-ALBI grade migration following TACE, of which 29.6% showed worsening EZ-ALBI grade. Unfortunately, worsening EZ-ALBI grade was not a significant predictive factor for survival, and the EZ-ALBI score following the procedure could not demonstrate prognostic ability among our patients. The different time points of the postprocedural evaluation could partly explain the different findings from the previous study. Since some patients might recover from recent decompensation, the dynamic change in liver function and EZ-ALBI grade at 1–3 months after TACE might not reflect actual patient survival [7, 22, 23]. Hence, pre-TACE EZ-ALBI scores should be used instead to provide an accurate and reliable prognostication.

Many predictive scores include total bilirubin and albumin levels as parameters in their formulas. CTP classification and MELD scores were popularly used to assess survival in patients with cirrhosis as well as patients with HCC [24]. The ALBI score, which eliminates subjective parameters of the CTP score, has been developed and has become a useful marker to assess the extent of liver impairment in patients with HCC [11, 25]. To avoid the complexity of ALBI calculation, the EZ-ALBI score has been proposed with a good correlation with the conventional ALBI score. In our study, the correlation between the EZ-ALBI and ALBI scores was excellent, with a correlation coefficient of 1.000, which was in line with the studies by Kariyama et al*.* and Ho et al [15, 16]. The prognostic performance of CTP, MELD, and ALBI scores among patients receiving TACE remains unclear in previous studies [26–29]. In this study, EZ-ALBI and ALBI scores showed high AUROCs and C-indices, which outperformed other models for anticipating death in this population. Moreover, the net benefit of EZ-ALBI and ALBI scores was higher than CTP and MELD scores for predicting one-, two- and three-year mortality. Because EZ-ALBI can be easily calculated, it is useful for bedside evaluations when a physician wants to make a quick mental calculation, and useful in situations where access to technology is limited.

Although recent studies reported that EZ-ALBI score had acceptable predictive power across all stages of HCC, only 23.3% of patients treated with TACE were included in the original cohort and the authors did not specifically explore the role of EZ-ALBI score in patients with intermediate-stage HCC [15]. Further, only 27.0% of patients undergoing TACE were enrolled in the Taiwanese validation set and the study reported a median OS of less than 25 months for patients treated with non-curative treatments, which comprised not only TACE but also systemic treatment and best supportive care [16]. With a small proportion of patients treated with TACE in previous studies, it could not make a firm conclusion for the prognostic performance of EZ-ALBI in patients with intermediate-stage HCC, which usually encompasses a highly heterogeneous population. Moreover, unlike the original Japanese cohort, which mainly comprised of patients with HCV infection (58.6%) [15], the patients in our study had HBV infection as the majority of liver disease. Data from a large cancer registry demonstrated more favorable survival in patients with HBV-associated HCC than in those with other causes of liver diseases [30]. Thus, a direct comparison of our cohort with the original study should be interpreted with caution.

To our knowledge, this is the first study to externally validate the EZ-ALBI score in patients treated with TACE. We confirmed that EZ-ALBI is a useful tool for stratifying patients with intermediate-stage HCC who undergo TACE. Our findings emphasize the important role of liver function assessment and optimal patient selection to maximize efficacy in patients treated with locoregional therapy.

Our study has several limitations. First, as a retrospective observational study, the possibility of unobserved confounding including selection and other biases cannot be discounted. Second, assessment of other relevant information, such as treatment for viral hepatitis and current viral status, was limited, and these data could impact patient survival. Third, our study was conducted in a single tertiary referral center, which may limit its generalizability to other settings. Lastly, although we included all patients meeting eligibility criteria, only 166 were available for the analysis. Despite this, the study endpoints were not sparse, allowing us to assess relationships with outcomes, and compare between prognostic scores. Nevertheless, external validation of our study findings in other settings, and with larger patient samples will be important to confirm the robustness of our study results.

## Conclusions

In conclusion, the EZ-ALBI score is an easy-to-calculate, reliable, and inexpensive stratifying biomarker of functional liver reserve, and a prognostic index for patients with intermediate-stage HCC treated with TACE. With its simplicity, EZ-ALBI may potentially replace the current complex prognostic scoring in real-life practice. Nevertheless, a large-scale prospective study to validate this novel score as a pre-TACE prognostic model to optimize patient benefits is warranted.

## Data Availability

The datasets generated and/or analysed during the current study are not publicly available due to the safety regulations from the hospital but are available from the corresponding author on reasonable request.

## Abbreviations

HCC: Hepatocellular carcinoma
TACE: Transarterial chemoembolization
EZ-ALBI: Easy albumin-bilirubin
ALBI: Albumin-bilirubin
BCLC: Barcelona clinic liver cancer
OS: Overall survival
HR: Hazard ratio
CI: Confidence interval
HBV: Hepatitis B virus
HCV: Hepatitis C virus
CTP: Child-Turcotte-Pugh
KCMH: King Chulalongkorn memorial hospital
AFP: Alpha-fetoprotein
SMA: Superior mesenteric artery
MELD: Model for end-stage liver disease
AUROC: Area under the receiver operating characteristic
C-index: Concordance index
IQR: Interquartile range
